# Mountains as Islands: Species Delimitation and Evolutionary History of the Ant-Loving Beetle Genus *Panabachia* (Coleoptera, Staphylinidae) from the Northern Andes

**DOI:** 10.3390/insects11010064

**Published:** 2020-01-20

**Authors:** Sofía I. Muñoz-Tobar, Michael S. Caterino

**Affiliations:** 1 Department of Plant and Environmental Sciences, Clemson University, Clemson, SC 29634, USA; mcateri@clemson.edu; 2 Escuela de Ciencias Biológicas, Pontificia Universidad Católica del Ecuador, Quito 1700525, Ecuador

**Keywords:** rove beetles, Coleoptera, speciation, Andes, páramo, Ecuador

## Abstract

The ant-loving beetle genus *Panabachia* Park 1942 is a poorly studied beetle lineage from the new world tropics. We recently collected *Panabachia* from several previously unrecorded locations in the páramo biome of the high Ecuadorian Andes, with males exhibiting great morphological variation in the distribution of the foveae and depressions in the pronotum, as well as aspects of the male genitalia. Here, we employ phylogenetic and species delimitation methods with mitochondrial (*COI*) and nuclear protein-coding (wingless) gene sequences to examine the concordance of morphological characters and geography with hypothesized species boundaries. Three methods of species delimitation (bPTP, GMYC and Stacey) were used to estimate the number of species, and divergence times between putative species using molecular clock calibration. Phylogenetic analysis revealed two parallel radiations, and species delimitation analyses suggest there are between 17 and 22 putative species. Based on clade support and concordance across species delimitation methods we hypothesize 17 distinct clusters, with allopatric speciation consistent with most geographic patterns. Additionally, a widespread species appears to be present in northern páramo sites, and some sister species sympatry may indicate other diversification processes have operated on certain lineages of *Panabachia*. Divergence time estimates suggest that *Panabachia* originated in the Miocene, but most species analyzed diverged during the Pliocene and Pleistocene (5.3–0.11 Mya), contemporaneous with the evolution of páramo plant species.

## 1. Introduction

The Andes mountain chain along the South American spine has a dynamic geological and climatological history. A wide range of geological processes, such as plate subduction, volcanism, crustal shortening and terrain accretion, has shaped the topography and the distribution of the species in the tropical Andes [1,2,3,4,5]. The orogenic formation of the Andes started during the Mesozoic and peaked with a massive uplift over the past 30 Ma [6]. The increase in elevation affected the climatic patterns of the region, leading to Quaternary formation of glaciers on the mountain summits [7,8]. 

These geological and paleoclimatical events have influenced tropical alpine ecosystems, as well as the distributions and genetic diversity of multiple evolutionary lineages that inhabit the tropical Andes, leading to high numbers of endemic species [3,9,10]. During interglacial fluctuations many suitable habitats moved from the mountain slopes into the inter-Andean valleys [5,11,12,13], sometimes allowing species to exchange genetic material between populations that were usually separated by elevation [5,14], or, alternately, driving the fragmentation of species distributions [5,13,15,16].

In the present day, a tropical alpine ecosystem known as páramo is found in the northern Andes above 2800 m, comprising numerous isolated island patches [17,18]. Multiple factors, including isolation due to elevation and climatic oscillations, have played into shaping the current diversity in the páramo [9,18]. Most species from páramo possess adaptations to live at high elevation [9]. These include morphological, physiological and behavioral adaptations as results of experiencing harsh abiotic conditions, such as extreme temperatures, higher solar radiation, desiccation and reduced oxygen pressure [19,20,21]. 

The phylogeographic structure of few Andean species has been assessed, most focusing on vascular plants and vertebrate species in a larger phylogeographical context [9,15,22,23]. These studies have revealed that most páramo lineages are quite young (0.0025–5.33 Mya–Pliocene and Pleistocene; [5]), and that the orogeny of the Andes has played an important role shaping their phylogeographical patterns [5,15,21,22,23]. The few studies done on insect lineages from high elevations have also shown that allopatric speciation is a contributing factor to their diversity patterns [24,25,26,27,28]. However, the specific patterns have varied depending on the dispersal capability of each insect lineage [27,28]. These studies have revealed some lineages to exhibit high levels of gene flow among populations, while others show higher genetic structure across páramo patches [27,28]. Basic evolutionary processes are not well understood for most alpine lineages [29], and the discrepancies among high elevation lineages analyzed to date offer distinct hypotheses that may be tested with insect lineages occurring in páramo.

The rove beetles (Coleoptera: Staphylinidae) represent one of the most diverse families of beetles (61,300 spp.) [30]. Their diversity has been attributed to the variety of habitats they inhabit, their feeding behaviors, and ecological interactions [31]. Many representatives of this family are found in the Neotropical Region, a region that is thought to contain one of the most diverse faunas of rove beetles [31]. In Ecuador, a total of 908 species of rove beetles have been reported [32], mainly from lowland areas. The general diversity of rove beetles in this region is thought to be much greater than previously documented [30], and mid- and high-elevation areas that present unique Andean microhabitats [33,34] are severely understudied.

In this study, we focused on diversification patterns in páramo populations of the genus *Panabachia* Park 1942, a Neotropical genus of ant-loving beetles (Coleoptera, Pselaphinae) that can be identified by the presence of a large trilobed excavation in the pronotum [35]. So far, only two species have been described within this genus: *P. vulnerata* (Sharp, 1887) from Panama, and *P. impressicollis* (Sharp 1887) from Guatemala [36,37,38]. However, this genus occurs across the Neotropical Region, from Mexico to Bolivia. Specimens have been reported from leaf litter as well as from bromeliads [38]. During the summer of 2016, *Panabachia* was collected from leaf litter samples taken in Ecuadorian páramo. Preliminarily, we have identified multiple morphospecies based on the distribution of foveae and depressions on the pronotum of the males, as well as aspects of the male genitalia (Figures S1 and S2). Still, a more comprehensive assessment of the morphological characters is needed, including more samples, considering only males in this genus appear to present diagnostic characters. In this study, we aim to investigate simultaneously the evolutionary history and species diversity in the genus *Panabachia* from páramo, addressing four specific questions: (1) How many evolutionarily independent lineages of *Panabachia* are present in the sampled material from páramo? (2) Are genetically isolated clades restricted to specific sites? (3) Is the distribution of genetic diversity limited by major geographic features such as rivers, dry valleys, and other subdivisions within the Ecuadorian Andes, as observed for some ground beetle lineages from páramo? and (4) Is the timing of diversification of *Panabachia* across páramo contemporary with establishment of Páramo in the high Andes (Miocene–Pliocene), or did it precede the current distribution of this ecosystem, as is apparent in some ground beetle lineages (e.g., *Pelmatellus columbianus*, 11.9 Mya and *Dyscolus alpinus*, 6.32 Mya [27,28]. These questions will be addressed through a combination of methods including species delimitation, phylogenetics and divergence time estimation.

## 2. Materials and Methods

### 2.1. Field Collection

**Figure 1:**
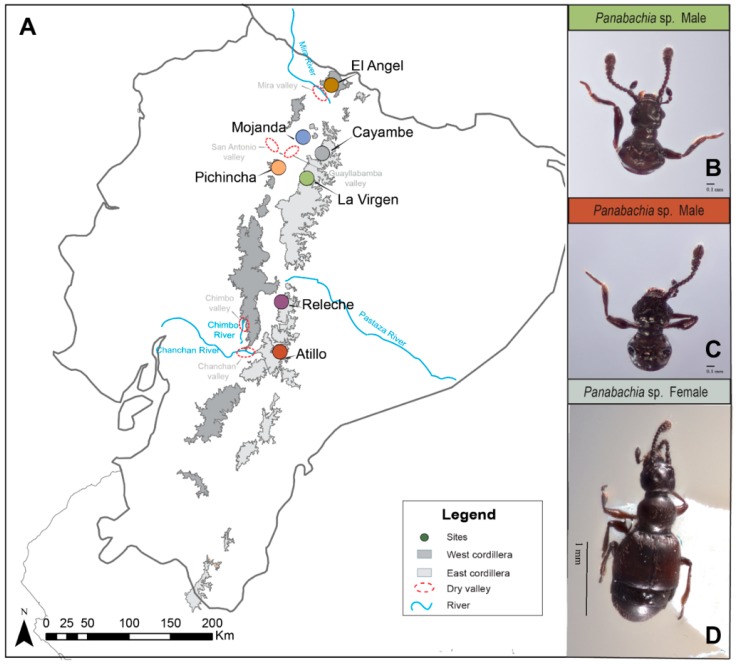
(**A**) Map of the collecting sites for *Panabachia* in the Ecuadorian Andes. Sites are represented by colored circles, the páramo ecosystem (above 3000 m) is highlighted in grey, and divided into East (light grey) and West (dark grey) cordillera. Potential geographical barriers (rivers and dry valleys) are highlighted in this map. (**B**) Head and pronotum from male individual from the La Virgen site. (**C**) Head and pronotum from male individual from the Atillo site. (**D**) Female individual from the Cayambe site.

Samples for this study were obtained from leaf litter samples from 7 sites across the highlands of Ecuador (Figure 1, Table 1). Three leaf litter samples were extracted per site, from a variety of litter types (*Polylepis* forest, moss, shrubs and grass). The selection of sites was based on conservation status, since most of the collecting took place within the network of protected areas. Collecting permits for this study were previously obtained (MAE-DNG-ARGG-CM-2014-004). The sifted material was transported to the lab and processed using Berlese funnels into 100% ethanol. Collected beetles were separated into morphospecies, based on characters examined (Figures S1 and S2). Wing size was also recorded for each specimen to understand their flight ability. Voucher specimens of this study will be deposited in the Museo de Zoología de la Pontificia Universidad Católica del Ecuador (QCAZ) after the study is concluded.

### 2.2. DNA Extraction, Amplification and Sequencing

The entire body of each beetle was used to extract genomic DNA using the GeneJet Genomic DNA Purification Kit (Thermo Fisher Scientific, Vilnius, Lithuania). Polymerase chain reaction was used to amplify two molecular markers: *COI* and *wingless*. The mitochondrial gene *COI* was amplified using the primers C1-J-2183 (5′-CAACATTTATTTTGATTTTTTGG-3′) and TL2-N-3014 (5′-TCCAATGCACTAATCTGCCATATTA-3′, [39]) following the amplification profile described by Caterino and Tishechkin (2014) [40]. For the nuclear gene *wingless*, we used the primers *wg*550f (5′-ATGCGTCAGGARTGYAARTGYCAYGGYATGTC-3′) and *wg*AbRZ (5′-CACTTNACYTCRCARCACCARTG-3′, [41]) following the amplification profile described by Parker and Grimaldi (2014) [42]. PCR reactions of 25 µL generally contained 2–3 µL genomic DNA, 17.5 μL water, 2.5 μL 10× buffer, 0.5 μL dNTPs, 0.75 μL MgCl_2_, 0.1 μL AmpliTaq^®^ DNA polymerase (Thermo Fisher Scientific, Waltham, MA, USA) and 1 μL of each primer (10 μm). Amplification cycles were performed in a Mastercycler^®^ nexus (Eppendorf). PCR products were purified using ExoSAP-IT (USB/Affymetric, Santa Clara, CA, USA), and sequencing was done commercially by Macrogen USA, Inc. (Rockville, MD, USA). Sequences were manually verified and trimmed using Geneious R8 (Biomatters Ltd., Auckland, New Zealand), and aligned using MAFFT v.7 (http://mafft.cbrc.jp/alignment/server/).

### 2.3. Phylogenetic Analyses

Models of molecular evolution were assessed using JModeltest 2.0 [43] for each molecular marker, where the GTR+I+G model appeared to be in the 100% confidence interval for *COI* and *wingless* data. The method of Templeton, Crandall and Sing (TCS) v1.21 [44] was used to construct haplotype networks for each data set. DnaSP V6.12 [45] was used to phase the *wingles*s data set. To reconstruct phylogenetic relationships among haplotypes RAxML version 8.2.8 [46] was launched from Mesquite’s Zephyr package [47], with 1000 bootstrap replicates. For Bayesian inference, we used Mr. Bayes 3.2 [48] through 20 million generations under default settings. 

Divergence time estimates were generated in BEAST 2.0 [49] using a partitioned two-gene data set. Two points of calibration were used, all designating minimum node age within the Pselaphinae: the first was an undescribed Bythinini from Burmese amber (99 Mya) [42]; the second was Parker and Grimaldi’s (2014) estimate that higher Pselaphinae arose 150 Mya. We employed an uncorrelated relaxed clock, using a log-normal distribution, and ran the analysis for 20 million generations. Output trees were generated using TreeAnnotator 2.0.02 (http://beast.bio.ed.ac.uk), with maximum clade credibility (MCC) after a 10% burn-in. 

### 2.4. Species Delimitation Analyses

The number of species within the genus *Panabachia* is unknown. Morphology-based species classification is a useful tool to determine species, yet the number of species can be masked by lack of species-diagnostic characters in one sex. Frequently in Pselaphinae, females do not exhibit diagnostic characters. Such is the case in *Panabachia* [36,50], and a high proportion of females were found among our samples collected. Therefore, we used three sequence-based species delimitation methods to hypothesize the number of reproductively isolated clades in *Panabachia* from páramo. The methods employed included: the Bayesian implementation of the Poisson tree processes (PTP) model, using the bPTP server (http://species.h–its.org/ptp/) [51]; a single threshold Generalized Mixed Yule Coalescent (GMYC) using the GMYC server (http://species.h–its.org/gmyc/; [52]; and a multi-species coalescent method, Species Tree And Classification Estimation Yarely (STACEY) v.1.2.4 [53], implemented in BEAST 2.0 [49]. Species delimitation analyses using these three models were performed using single locus and multilocus data sets. 

For analyses in bPTP, trees generated in Mr. Bayes were used as input. Analyses were run through 100,000 MCMC generations, with a thinning of 100 and 10% burn-in. For GMYC, ultrametric trees were produced in BEAST 2.0 [49], using an uncorrelated relaxed clock, a constant coalescent speciation process prior, through 10,000,000 generations and 10% burn-in. Effective Sample Size (ESS) was evaluated in Tracer v1.5 [54], considering runs with ESS values above 200. Output trees were generated in TreeAnnotator 2.0.02 (http://beast.bio.ed.ac.uk), using maximum clade credibility (MCC) after a 10% burn-in and median heights for node heights. Resulting trees were used as input in the GMYC server, using a single threshold. Lastly, for the implementation of STACEY in BEAST 2.0 [49], files were generated in BEAUTI v.2.4.0 [49]. For the minimal number of clusters, two scenarios were analyzed from all taxa’s specimens divided as different species to clusters defined by site. The epsilon value was set to 1 × 10^−4^, following guidelines in the software documentation; nucleotide substitution models were estimated a priori using PAUP 4.0 [55]; a fossilized birth-death model was selected for speciation; and the uncorrelated lognormal model was used to describe the relaxed molecular clock. Input files were run for 500 million iterations, sampling every 10,000th generation. Two replicates were run for each set, ESS values were evaluated in Tracer v.1.6, and independent runs were combined using LogCombiner v.2.4.0 [49] after a 10% burn-in; output trees were summarized in TreeAnnotator [49]. 

## 3. Results

### 3.1. Sequence Data and Polymorphisms

**Figure 2:**
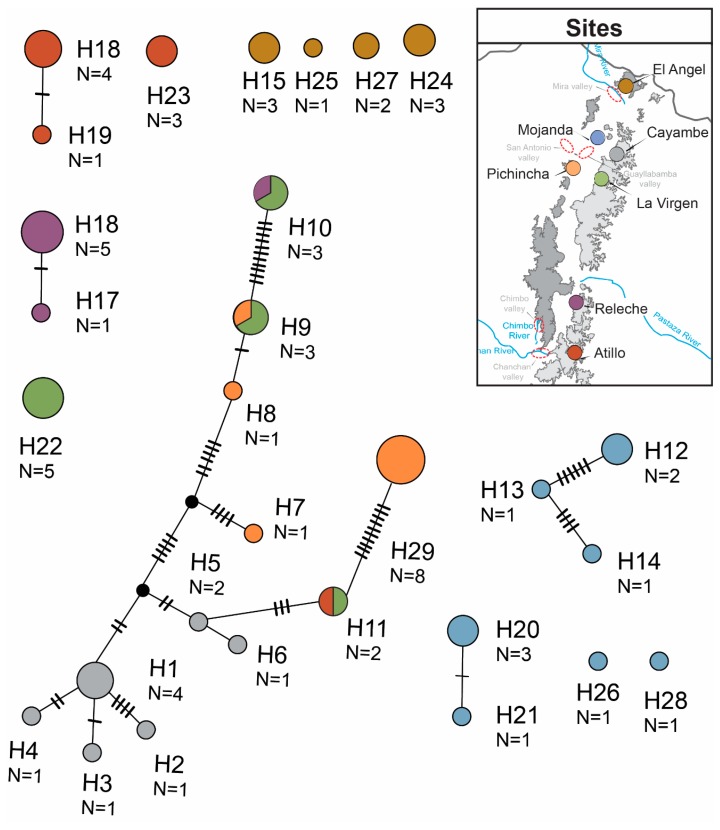
TCS haplotype networks for the *COI* gene of *Panabachia*.

**Figure 3:**
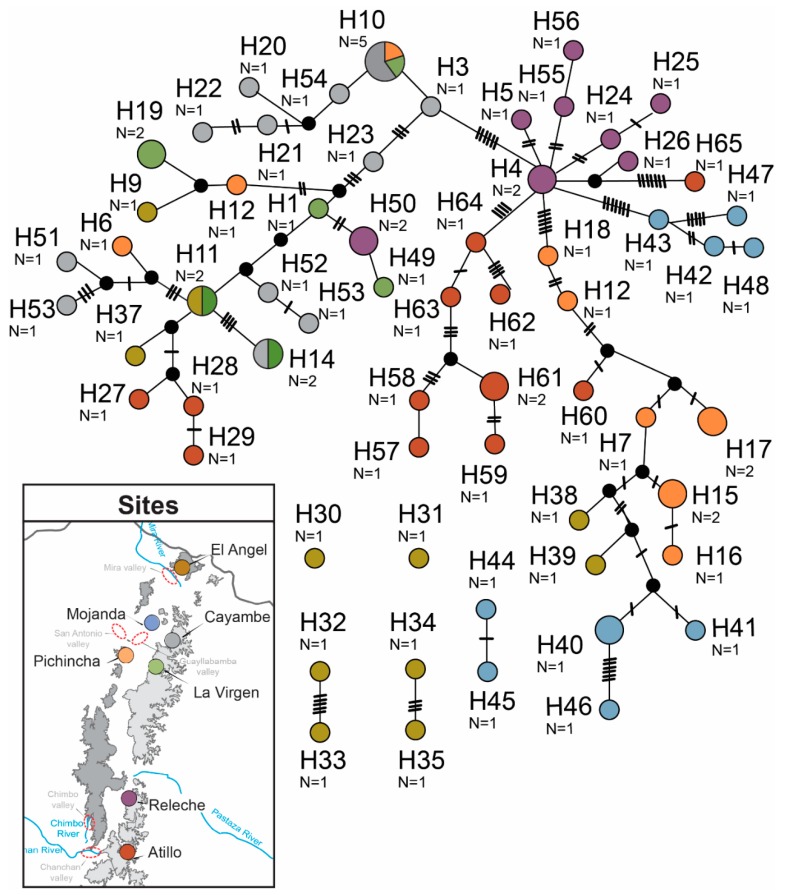
TCS haplotype networks for the nuclear gene, *wingless* of *Panabachia*.

Sampling from litter resulted in the collection of 68 adult *Panabachia* from seven localities (Figure 1). For phylogenetic analyses, we used 10 individuals per site where possible (Table 1), although Releche only yielded seven individuals. The *COI* gene was amplified from 67 samples (GenBank accessions MN536369 to MN536434, Table 2), and the alignment of this gene had a total of 765 base pairs. Of the 765 base pairs, 240 were variable, 66 were parsimony informative (Table 3), and 30 distinct haplotypes were identified using TCS (Figure 2). The *wingless* gene was amplified from 62 individuals (GenBank accessions MK674898 to MK674959, Table 2). The alignment for this gene had 445 base pairs with 108 segregating sites from which 105 were parsimony informative (Table 3). A total of 65 alleles were identified in the *wingless* data set (Figure 3). Phased data showed that most individuals are homozygotic, with only 16 heterozygotic individuals. Amongst the two data sets, only a few haplotypes/alleles were shared among sites. Individuals from La Virgen shared *COI* haplotypes with individuals from Pichincha (H9), Atillo (H11) and Releche (H10) (see Figure 2). For the *wingless* data set, three alleles are shared among sites. One allele is shared by the northern populations of La Virgen, Cayambe and Pichincha (H10), and La Virgen shares alleles with el Angel (H11) and Cayambe (H15; Figure 3). The overall nucleotide diversity for each data set was low (wingless: π = 0.047, *COI*: π = 0.113; Table 3).

### 3.2. Species Delimitation Analyses

**Figure 4:**
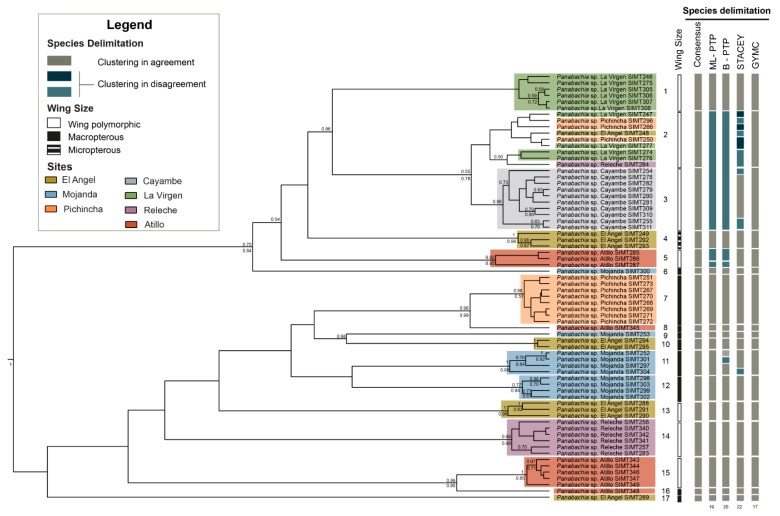
Bayesian inference based on a combined data set for *Panabachia* putative species. Colored bars represent site information, and species delimitation analysis using bPTP, STACEY and GMYC are represented by bars on the right side of the phylogeny. The grey color on the bars signifies there is an agreement across methods of delimitation, while the color blue signifies that there is disagreement across methods, and the two shades of blue signify that they belong to the same group within each clade. Wing size is represented in bars, white colored bars indicate wing polymorphic clades (micropterous and macropterous), black bars macropterous clades, and the black and white bar represents a micropterous clade. Posterior probabilities are shown above the branches and RAxML bootstrap values are shown below branches. Branch lengths are not proportional with the number of changes.

Results from single locus and multilocus analyses using three models of species delimitation are summarized in Figure 4. Multilocus analyses identified 17–22 putative species, with a high level of congruence for most groups. The single locus analyses, however, showed a high variation among outputs. *Wingless* showed a wide range of results depending on the method used: STACEY suggested 62 clades (out of 68 individuals); bPTP showed 51 clades but GMYC suggested only three species-level clusters (Figure S3). For the mitochondrial gene, results from bPTP and STACEY showed a higher level of correspondence with results from the multilocus analyses, with 20 clades identified in bPTP and 22 in STACEY. This was not seen in the GMYC analysis, where only four clades were observed (Figure S1). 

For further interpretation of phylogenetic relationships and geographical distribution of genetic clusters, a conservative number of putative species (17) will be considered the most reasonable hypothesis (Figure 4). This hypothesis was based in part on the statistical support for each lineage in the phylogenetic inferences and the distribution of haplotypes in the TCS network analysis. We also assume that some splits observed in bPTP and STACEY multilocus analyses probably represent intraspecific variation; for example, for clade 11 from Mojanda (bPTP) and clade 3 from Cayambe (STACEY). This suggestion is based on the low number of mutations among these haplotypes in TCS analyses. This number of presumed species also corresponds well with preliminary morphological analyses: characters in the pronotum of the male, such distribution of the foveae and shape of the pronotum, show correspondence with the proposed groups (Figures S1 and S2). As of yet, not all putative species are represented by male specimens, so complete correspondence cannot be assessed. We were also able to trace the presence and absence of wings to assess potential dispersal ability for each proposed clade (Figure 4). We found that most clades are represented by macropterous specimens (clade 6–12 and 16–17). However, numerous wing polymorphic clades are found (clade 1–3, 5 and 13–15), where males are macropterous and females are micropterous. One clade was represented only by micropterous individuals (clade 4).

### 3.3. Phylogenetic Analyses and Divergence Time Estimates 

**Figure 5:**
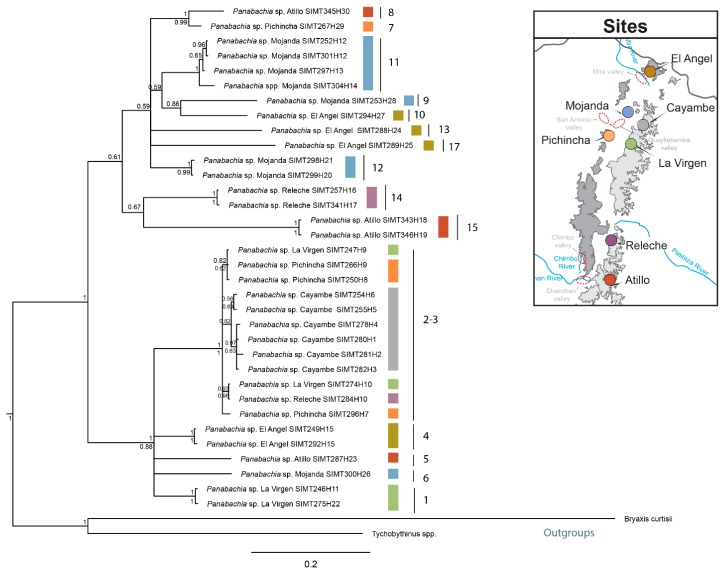
Posterior probability tree for the mitochondrial gene *COI* of *Panabachia*. Branch lengths are in proportion to the number of substitutions per site, in reference to the scale bar. Posterior probabilities are shown above the branches and bootstrap support values for the Maximum Likelihood tree are shown below branches.

**Figure 6:**
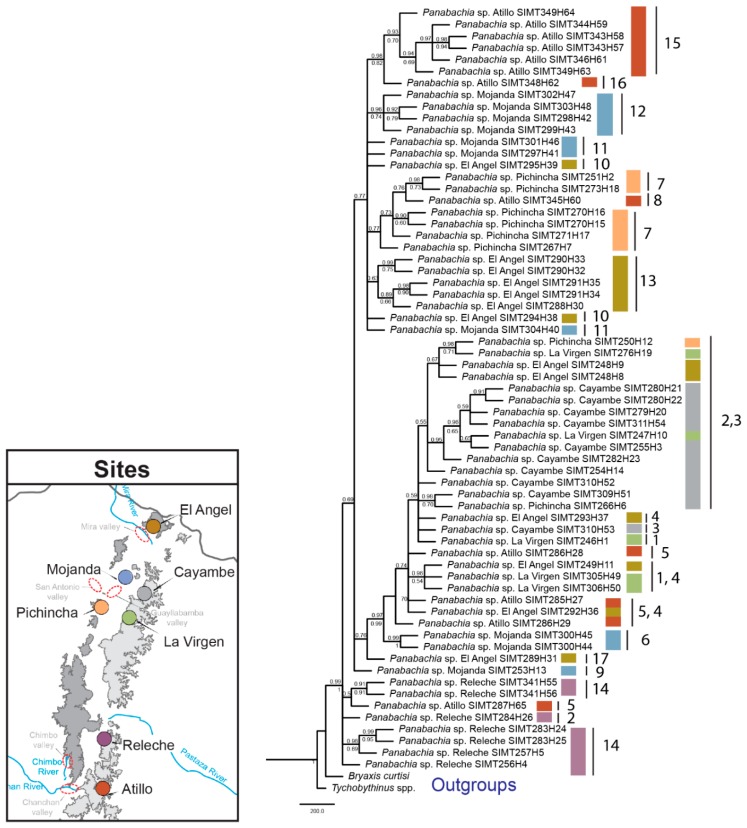
Posterior probability tree for the nuclear protein-coding gene wingless of *Panabachia*. Branch lengths are in proportion to the number of substitutions per site, in reference to the scale bar. Posterior probabilities are shown above the branches and bootstrap support values for the ML tree are shown below branches.

Phylogenetic inferences generated through Maximum Likelihood and Bayesian methods show two separate clades in the combined data set tree as well for the *COI* gene tree (Figure 4 and Figure 5). Most putative species identified through species delimitation analyses appear to be well supported by bootstrap and posterior probability values (Figure 4). Exceptions are found for clades 1, 2 and 17, which do not have strong branch support (Figure 4). Gene trees did not recover all the genetic clusters observed in the species delimitation analyses (Figure 5 and Figure 6). For example, the *COI* gene tree shows strong bootstrap and posterior probability values for most putative species (Figure 5). But clades 2 and 3 are exceptions. In the case of the *wingless* gene tree, bootstrap and posterior probability values only support seven clades out of the 17 proposed (Figure 6), and multiple incongruences were found in this gene, contributing to the lower phylogenetic resolution, where clades 2, 3, 4, 5, 7, 11, and 14 appear at multiple points. In contrast, the *COI* tree only presented one incongruence, for individuals of clade 2. The *COI* tree topology largely resembles the combined data set result, where clade 2 is resolved as a single clade (Figure 4). 

In reference to the distribution of putative species, clear geographical splits among sites were not detected in the phylogeny, since multiple species were identified for the majority of sites (e.g., four clades in Mojanda, Figure 4). Furthermore, the two main clades both include representatives across most of the area sampled (Figure 4). However, at a finer scale, some sister clades do show interesting allopatric disjunctions. This was the case of clade (Pichincha, W) and 7 (Atillo, E), separated by distance and side of the mountain range. The separation between clades 9 (Mojanda, W) and 10 (El Angel, W), separated by <70 km, spans the dry Mira River valley. 

**Figure 7:**
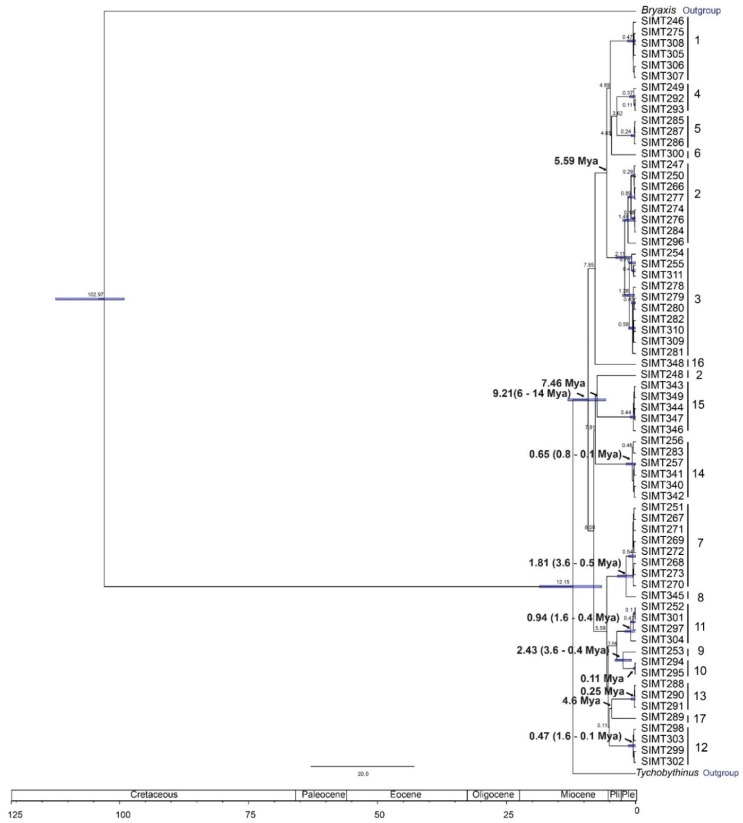
Divergence time estimation for *Panabachia* based on a relaxed molecular clock, using a combined data set.

Divergence time estimates suggest that *Panabachia* in Ecuadorean páramo originated in the Miocene (9.2 Mya, Figure 7), and that most of the proposed species diverged during the Pliocene and Pleistocene (5.3–0.11 Mya). Two parallel radiations are observed through phylogenetic analyses; these radiations started in the Miocene (8–7.85 Mya) and continued throughout the Pleistocene. The first radiation event (7.86–0.47 Mya) gave rise to clade 7–17, and its sister gave rise to clade 1–6 (4.65–0.24 Mya). This second radiation is composed mostly of northern groups (clades 1–4, and 6), with the exception of clade 5, represented by specimens collected in Atillo (SE). Clade 2 was also exceptional, in that it contained individuals from several sites (La Virgen, Pichincha, El Angel and Releche), the last of which is quite remote from the others. 

## 4. Discussion

High elevation species are particularly interesting given the climatic diversity, high levels of isolation and complex geological history of mountain systems [4,9,56,57]. Yet, alpine beetle faunas from the Andes have only been superficially explored. Previous work in the Ecuadorian páramo has shown distinct patterns of genetic distribution in ground beetles, from higher population structure in flightless ground beetles [28] to high levels of genetic connectivity between populations of a macropterous species [27]. Still, the genetic diversity of other alpine beetle lineages from the Andes has not been assessed, leaving the question open as to whether other alpine insect lineages are following similar patterns as the ground beetles. 

Species delimitation analyses facilitate the identification of distinct evolutionary lineages within a sample of individuals [51,52,58], and the use of multilocus genetic data has proven to be a powerful tool for delimiting species [58]. Yet, methods to delimit species vary greatly in parameters and outcomes, and the search for congruence across results from species delimitation models can provide more reliable hypotheses for species boundaries. Results from the species delimitation analyses show that *Panabachia* from páramo comprises a diversity of species; 17–22 putative species were identified with bPTP, STACEY and GMYC using a multilocus data set. The three models of species delimitation showed similar outcomes for most species clades (Figure 4). Clustering disagreement was only reported in four clades, where bPTP and STACEY tended to subdivide lineages more finely, showing 1–5 more clades than GMYC. Evidence from phylogenetic inferences suggests that some delimited species using bPTP and STACEY might actually represent intrapopulation variation. This seems to be the case for clade 11 from Mojanda, which is divided by bPTP into two species lineages. In clade 5, from Atillo, bPTP also recognizes two presumed species. This appears be the result of the occurrence of highly divergent genotypes in the *wingless* data set (3 haplotypes in the *wingless* data set vs 1 haplotype in the *COI* data set). For clades 2 and 3, a total of one, two, or four species clades are recognized, depending on algorithm (bPTP, GMYC, or STACEY, respectively). Based on clade support and distribution of haplotypes in TCS, these individuals appear to represent two distinct species clusters. 

More broadly, páramo *Panabachia* appear to represent two parallel radiations, at least by COI and the combined data (Figure 4 and Figure 5), though relationships within each are not clearly resolved. Divergence time estimates show that these radiations started during the Miocene (5.59–7.81 Mya), but 14 out of 17 putative species of *Panabachia* from páramo originated during the Pleistocene (0.11–4.6 Mya, Figure 7). These estimates are contemporary with the environment they live in, since the Andes reached its current elevation during the Pleistocene [1]. The increase in elevation created suitable conditions for the development of high elevation species [59], which are thought to have evolved from closely related lineages from the lowland tropical areas, as well as from lineages from temperate regions [9,60,61]. Studies of plant lineages from páramo also show accelerated rates of diversification during this period of time [9]. 

The main factors that affect cladogenetic events are associated with geographical isolation or ecological shifts [62]. Although most of the genetic clusters of *Panabachia* are well supported by bootstrap and posterior probability values, and represent distinct geographical areas, few phylogenetic relationships between clades are adequately supported to connect to geological events or features. Some correspondence with geography was found in the *COI* gene tree (Figure 5). For example, the split between species 8 and 7 spans opposite sides of the mountain range (Atillo and Pichincha). However, most of the speciation events with well supported branches show divergence between presumed species on the same sides of the mountain ranges but separated by distance (clades 9–10 and 14–15). In some instances, for example between clade 9 and 10, the distance might be reinforced by the presence of a putative barrier (Mira river and valley). 

While allopatric speciation might explain some of the patterns in *Panabachia* from páramo, it does not explain the high number of sympatric clades found in El Angel (4), Mojanda (4) and Atillo (3). Climatic oscillation during the Pleistocene glaciations and topographic characteristics of each cordillera appear to influence the level of connectivity and fragmentation of populations across the northern Andes [5]. Understanding the timing of local geological events might give us insight into the factors that influence diversity in these sites; for example, volcanic activity dates to 10,000 years BP in Mt. Mojanda and Mt. Fuya in Mojanda, and Mt. Chiles in El Angel [63,64]. For Atillo, evidence for the presence of small glaciers during the Pleistocene and Holocene was found on Mt. Ayapungo (4730 m) and Mt. Coyay (4630 m) [65], mountains that are adjacent to Atillo. The increased number of putative species of these particular sites could be the result of multiple re-colonization events from adjacent areas during recent environmental fluctuations, as seen in other mountain systems [66,67]. 

Apart from the effect of environmental conditions and ecological interactions, the dispersal ability of each beetle lineage plays an important role in species diversification [28,68,69,70]. From previous studies done in ground beetles from páramo, we understand that the loss or reduction of wings can promote diversification events for some beetle lineages [28]. This appears not to be a factor for most putative species of *Panabachia* from páramo, where more than half of the proposed species are macropterous (56%, Figure 4). Still, wing polymorphic species of *Panabachia* represent a substantial proportion of the assessed clades (41%, Figure 4), where males are macropterous and females are micropterous or brachypterous. Yet, distinct wing morphologies are not restricted to a geographical area, and both wing polymorphic and macropterous groups can be found in the same sites. Further sampling and a better understanding of the distributional range and natural history of each species is needed to assess whether dispersal ability is a significant driver of diversification of this beetle lineage.

The high diversity found in *Panabachia* might be associated with the high diversity of leaf litter types sampled. These included decomposing grass leaves and roots, *Polylepis* and Compositae leaf litter, and moss over rocks and rotten wood. This microhabitat diversity is related to the high diversity of plants and plants forms (from cushion plants and shrubs, to herbaceous rosettes) páramo presents [60,71]. However, microhabitat-focused sampling has not been systematic enough to measure correspondence of putative species of *Panabachia* with particular types of leaf litter. More focused sampling and more information about the natural history of the group will be needed to determine if any such associations are related to diversification rates.

In comparison with patterns found among ground beetles from páramo, *Panabachia* from páramo diverged in more recent times (mostly in the Pleistocene). Most ground beetle species from Ecuadorian páramo evolved during the Miocene, prior to the evolution of this ecosystem (~6–20 Mya) [27,28]. Phylogeographic breaks in *Panabachia* are not as clear as in the *Dercylus* lineage (Carabidae, Harpalinae), where the presence of geographical barriers (e.g., rivers, dry valleys and mountain range) had a great effect on the pattern of speciation of this group [28]. Yet, most of the proposed species within the *Panabachia* lineage do represent restricted geographical areas. 

When the diversity of *Panabachia* is compared to the widely distributed *Pelmatellus columbianus* (11.19 Mya, Carabidae, Harpalinae), clade 2 of *Panabachia* show similar patterns in the distribution of the genetic diversity, since members of this clade are present across multiple sites. Preliminary analyses of the population structure of species 2 (not shown), suggest Releche represents a distinct genetic cluster. We found incongruence among gene trees for an individual from Releche (SIMT284), which in the *COI* gene tree is grouped with clade 2, while in the *wingless* tree is found as sister to clade14. Hence, a more comprehensive analysis will require additional samples from each population to determine if this clade represents one or two proposed species. Nevertheless, widely distributed species of ground beetles have been reported at the same sites as this widespread clade of *Panabachia*. Such is the case in *Pelmatellus columbianus* (Cayambe, La Virgen, Pichincha, Releche) and *Dyscolus alpinus* (Cayambe, Pichincha, La Virgen [28], which suggests similar factors are affecting the distribution of these northern beetle lineages. In particular, the effect of Quaternary glaciation might have enabled gene flow between sites now isolated by elevation [14]. 

The study of multiple beetle lineages from páramo is slowly providing a better understanding or the evolution of high elevation beetle faunas. An increasing number of studies have concentrated on ground beetle species from the Ecuadorian Andes [17,72,73]. Other beetle lineages from páramo have been less studied, but results to date show that high elevation faunas tend to have an elevated number of endemic species [14,17,72,73,74]. Numerous putative species of *Panabachia* (those documented herein as well as others) have yet to be described, further underscoring the importance of conserving high elevation ecosystems. Although most of the sampled sites are already protected areas [75], many high elevation areas across the Ecuadorian Andes are not part of this network of national parks. 

## 5. Conclusions

Overall, *Panabachia* represents a promising model for the study of diversification of beetles from high elevation areas in the Andes, considering its high interpopulational diversity and recent divergence (Pliocene and Pleistocene, 5.3–0.11 Mya). Through the use of three methods of species delimitation analyses and two molecular markers we were able to identify 17 putative species from seven sites in the Ecuadorian Andes. Assessed species appear to be restricted to small geographical ranges, with exception of one species clade present in multiple sites in the northern Ecuadorian Andes. The distribution of genetic diversity of *Panabachia* is complex, and a generalized pattern for alpine beetles of the Ecuadorian Andes has yet to emerge. Multiple factors appear to be shaping the genetic diversity of this beetle lineage, such as mountain isolation, habitat discontinuity and dispersal capability. The pattern of divergence observed across the tree topologies in this study certainly does not capture the entire genetic diversity of the *Panabachia* lineage, since sampling was only focused on isolated páramo patches. Further sampling (especially cloud and montane forest) and more information about the natural history of the species are needed to develop a more comprehensive picture of the distribution of phylogenetic diversity of *Panabachia*. The present study should provide a strong preliminary framework for more thorough systematic treatment of this diverse genus.

## Figures and Tables

**Table 1:** Population and site information for each sample of *Panabachia* from páramo sites.

Mountain Range	No.	Site	N	Latitude	Longitude	Elevation	Collecting Date
West	1	El Angel	10	00°42.3521′ N	77°57.985′ W	3301 m	26 July 2016
	2	Mojanda	10	00°08.710′ N	78°16.753′ W	3715 m	12 July 2016
	3	Pichincha	11	00°11.259′ S	78°32.432′ W	3897 m	22 June 2016
East	4	Cayambe	10	00°02.101′ S	78°03.608′ W	3743 m	1 June 2016
	5	La Virgen	10	00°18.477′ S	78°13.953′ W	3694 m	28 June 2016
	6	Releche	7	01°38.400′ S	78°30.426′ W	3124 m	8 July 2016
	7	Atillo	10	02°11.265′ S	78°31.2601′ W	3501 m	7 July 2016

**Table 2:** GenBank accession numbers for voucher specimens.

Voucher ID	Genus	Species	Site	Haplotype	*COI*	Haplotype	*Wingless*	Reference
SIMT248	*Panabachia*	sp.	El Angel	–	–	H8/H9	MK674905	This study
SIMT249	*Panabachia*	sp.	El Angel	H15	MN536380	H11	MK674907	This study
SIMT288	*Panabachia*	sp.	El Angel	H24	MN536401	H30	MK674927	This study
SIMT289	*Panabachia*	sp.	El Angel	H25	MN536402	H31	MK674928	This study
SIMT290	*Panabachia*	sp.	El Angel	H24	MN536403	H32/H33	MK674929	This study
SIMT291	*Panabachia*	sp.	El Angel	H24	MN536404	H34/H35	MK674930	This study
SIMT292	*Panabachia*	sp.	El Angel	H15	MN536405	H36	MK674931	This study
SIMT293	*Panabachia*	sp.	El Angel	H15	MN536406	H37	MK674932	This study
SIMT294	*Panabachia*	sp.	El Angel	H27	MN536407	H38	MK674933	This study
SIMT295	*Panabachia*	sp.	El Angel	H27	MN536408	H39	MK674934	This study
SIMT252	*Panabachia*	sp.	Mojanda	H12	MN536371	–	–	This study
SIMT297	*Panabachia*	sp.	Mojanda	H13	MN536410	H40/H41	MK674936	This study
SIMT298	*Panabachia*	sp.	Mojanda	H21	MN536411	H42	MK674937	This study
SIMT299	*Panabachia*	sp.	Mojanda	H20	MN536412	H43	MK674938	This study
SIMT300	*Panabachia*	sp.	Mojanda	H26	MN536413	H44/H45	MK674939	This study
SIMT301	*Panabachia*	sp.	Mojanda	H12	MN536414	H46	MK674940	This study
SIMT302	*Panabachia*	sp.	Mojanda	H20	MN536415	H47	MK674941	This study
SIMT303	*Panabachia*	sp.	Mojanda	H20	MN536416	H48	MK674942	This study
SIMT304	*Panabachia*	sp.	Mojanda	H14	MN536417	H40	MK674943	This study
SIMT253	*Panabachia*	sp.	Mojanda	H28	MN536382	H13	MK674909	This study
SIMT250	*Panabachia*	sp.	Pichincha	H8	MN536381	H12	MK674908	This study
SIMT251	*Panabachia*	sp.	Pichincha	H29	MN536370	H2	MK674899	This study
SIMT266	*Panabachia*	sp.	Pichincha	H9	MN536375	H6	MK674903	This study
SIMT267	*Panabachia*	sp.	Pichincha	H29	MN536376	H7	MK674904	This study
SIMT268	*Panabachia*	sp.	Pichincha	H29	MN536377	–	–	This study
SIMT269	*Panabachia*	sp.	Pichincha	H29	MN536378	–	–	This study
SIMT270	*Panabachia*	sp.	Pichincha	H29	MN536384	H15/H16	MK674911	This study
SIMT271	*Panabachia*	sp.	Pichincha	H29	MN536385	H17	MK674912	This study
SIMT272	*Panabachia*	sp.	Pichincha	H29	MN536386	H17	MK674913	This study
SIMT273	*Panabachia*	sp.	Pichincha	H29	MN536387	H15/H18	MK674914	This study
SIMT296	*Panabachia*	sp.	Pichincha	H7	MN536409	H10	MK674935	This study
SIMT254	*Panabachia*	sp.	Cayambe	H6	MN536383	H14	MK674910	This study
SIMT255	*Panabachia*	sp.	Cayambe	H5	MN536372	H3	MK674900	This study
SIMT278	*Panabachia*	sp.	Cayambe	H4	MN536392	–	–	This study
SIMT279	*Panabachia*	sp.	Cayambe	H1	MN536393	H10/H20	MK674919	This study
SIMT280	*Panabachia*	sp.	Cayambe	H1	MN536394	H21/H22	MK674920	This study
SIMT281	*Panabachia*	sp.	Cayambe	H2	MN536395	H10	MK674921	This study
SIMT282	*Panabachia*	sp.	Cayambe	H3	MN536396	H23	MK674922	This study
SIMT309	*Panabachia*	sp.	Cayambe	H1	MN536422	H51	MK674947	This study
SIMT310	*Panabachia*	sp.	Cayambe	H1	MN536423	H52/H53	MK674948	This study
SIMT311	*Panabachia*	sp.	Cayambe	H5	MN536424	H10/H54	MK674949	This study
SIMT246	*Panabachia*	sp.	La Virgen	H11	MN536369	H1	MK674898	This study
SIMT247	*Panabachia*	sp.	La Virgen	H9	MN536379	H10	MK674906	This study
SIMT274	*Panabachia*	sp.	La Virgen	H10	MN536388	H11	MK674915	This study
SIMT275	*Panabachia*	sp.	La Virgen	H22	MN536389	H14	MK674916	This study
SIMT276	*Panabachia*	sp.	La Virgen	H10	MN536390	H19	MK674917	This study
SIMT277	*Panabachia*	sp.	La Virgen	H9	MN536391	H19	MK674918	This study
SIMT305	*Panabachia*	sp.	La Virgen	H22	MN536418	H49	MK674944	This study
SIMT306	*Panabachia*	sp.	La Virgen	H22	MN536419	H50	MK674945	This study
SIMT307	*Panabachia*	sp.	La Virgen	H22	MN536420	H50	MK674946	This study
SIMT308	*Panabachia*	sp.	La Virgen	H22	MN536421	–	–	This study
SIMT256	*Panabachia*	sp.	Releche	H16	MN536373	H4	MK674901	This study
SIMT257	*Panabachia*	sp.	Releche	H16	MN536374	H5	MK674902	This study
SIMT283	*Panabachia*	sp.	Releche	H16	MN536397	H24/H25	MK674923	This study
SIMT284	*Panabachia*	sp.	Releche	H10	MN536398	H26	MK674924	This study
SIMT340	*Panabachia*	sp.	Releche	H16	MN536425	–	–	This study
SIMT341	*Panabachia*	sp.	Releche	H17	MN536426	H55/H56	MK674950	This study
SIMT342	*Panabachia*	sp.	Releche	H16	MN536427	H4	MK674951	This study
SIMT285	*Panabachia*	sp.	Atillo	H23	MN536399	H27	MK674925	This study
SIMT286	*Panabachia*	sp.	Atillo	H23	MN536400	H28/H29	MK674926	This study
SIMT287	*Panabachia*	sp.	Atillo	H23	MN536434	H65	MK674959	This study
SIMT343	*Panabachia*	sp.	Atillo	H18	MN536428		MK674952	This study
SIMT344	*Panabachia*	sp.	Atillo	H18	MN536429	H59	MK674953	This study
SIMT345	*Panabachia*	sp.	Atillo	H30	MN536430	H60	MK674954	This study
SIMT346	*Panabachia*	sp.	Atillo	H19	MN536431	H61	MK674955	This study
SIMT347	*Panabachia*	sp.	Atillo	H18	MN536432	H61	MK674956	This study
SIMT348	*Panabachia*	sp.	Atillo	–	–	H62	MK674957	This study
SIMT349	*Panabachia*	sp.	Atillo	H18	MN536433	H63/H64	MK674958	This study
–	*Bryaxis*	*curtisi*	Outgroup	–	KM350460	–	KM350297	Parker and Grimaldi (2014).
–	*Tychobythinus*	sp.	Outgroup	–	KM350498	–	KM350290	Parker and Grimaldi (2014).

**Table 3:** Overall genetic indexes for the genus *Panabachia*. N refers to the number of individuals sampled; S, number of segregating sites; Ps, number of parsimony informative sites; π, is a measure of nucleotide diversity; θ is a measure of genetic diversity; D represents Tajima’s D, a neutrality test.

Gene	N	S	Ps	θ	Π	D
*COI*	67	240	66	0.054	0.113	1.73 (*p* > 1.78)
*wingless*	62	118	105	0.056	0.047	−0.51 (*p* > 0.10)

## Supplementary Materials

The following are available online at https://www.mdpi.com/2075-4450/11/1/64/s1, Figure S1: Male morphological characters for clades 1 and 5, showing high morphological variation; Figure S2: Male morphological characters for clades 1 and 5, showing high morphological variation Figure S3: Single locus and multilocus species delimitation analyses for *Panabachia*.
