# Supplementary figures and images for: Mountains as Islands: Species Delimitation and Evolutionary History of the Ant-Loving Beetle Genus Panabachia (Coleoptera, Staphylinidae) from the Northern Andes
Source: Insects. 2020 Jan 20;11(1):64. doi: 10.3390/insects11010064 (PMC7023032; doi:10.3390/insects11010064)

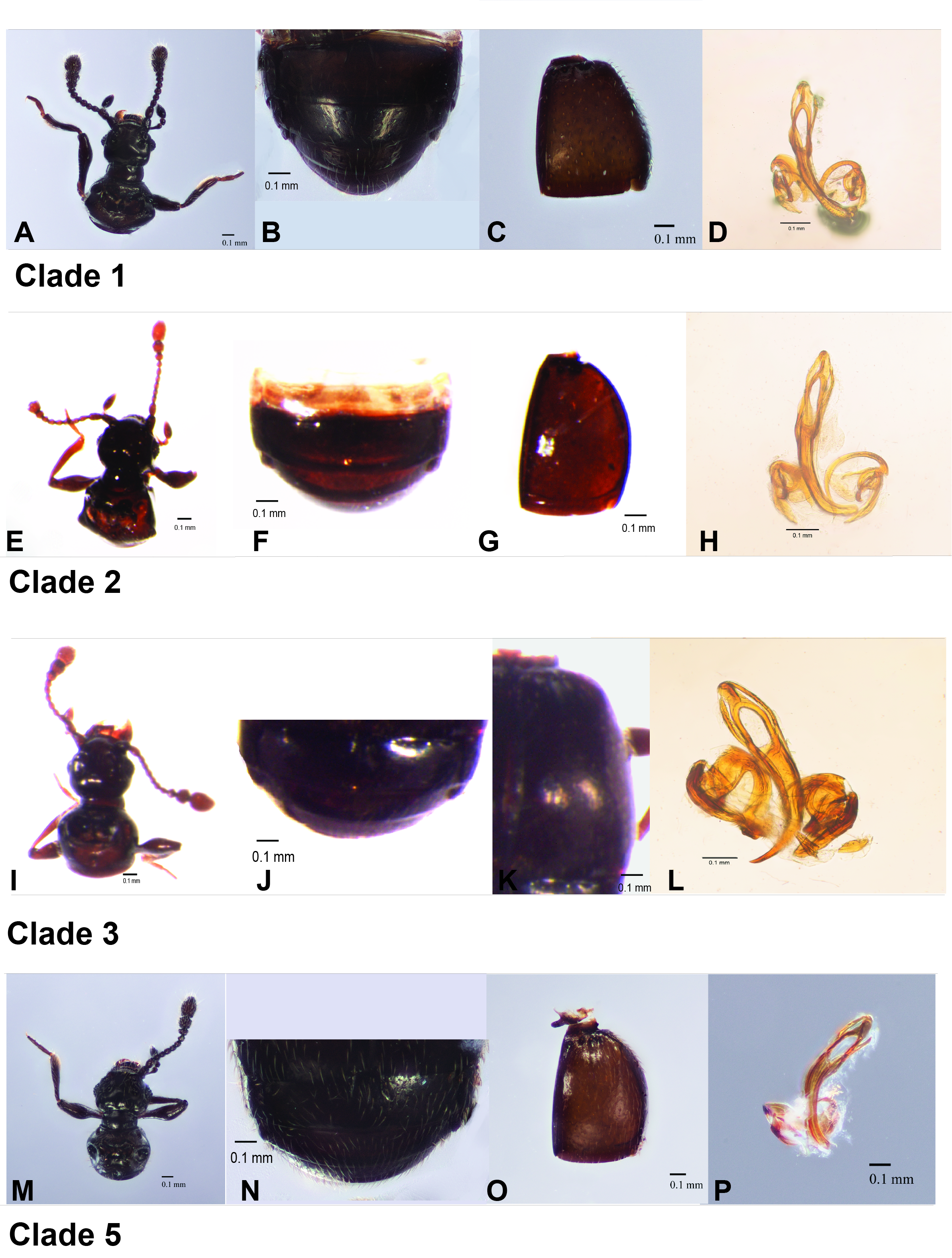

Supplement: Supplementary file 1 [file insects-11-00064-s001.zip › SF1.tif]

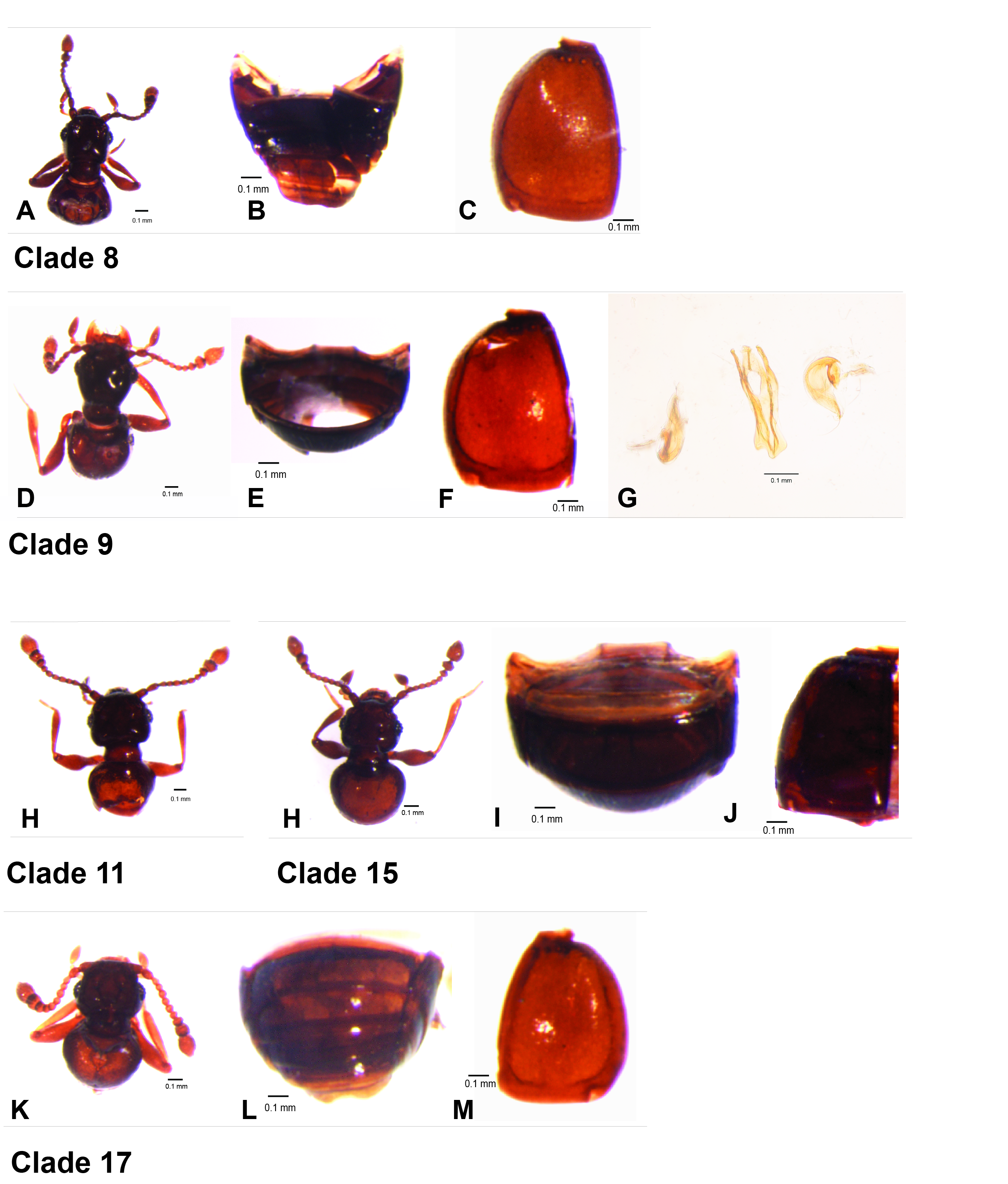

Supplement: Supplementary file 1 [file insects-11-00064-s001.zip › SF2.tif]

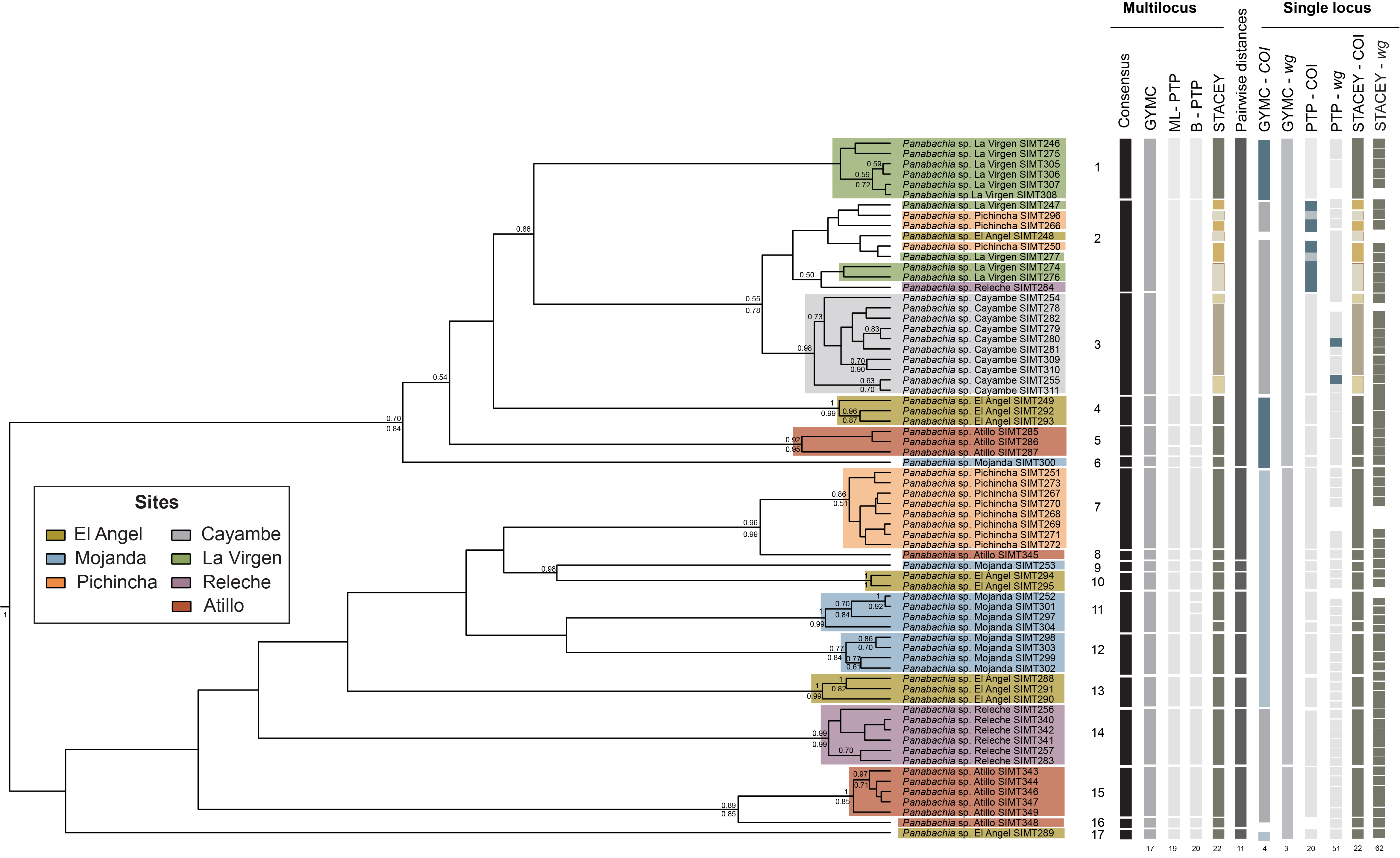

Supplement: Supplementary file 1 [file insects-11-00064-s001.zip › SF3.tif]
